# A review of the methodological features of systematic reviews in maternal medicine

**DOI:** 10.1186/1741-7015-5-10

**Published:** 2007-05-24

**Authors:** Lumaan Sheikh, Shelley Johnston, Shakila Thangaratinam, Mark D Kilby, Khalid S Khan

**Affiliations:** 1 Academic Unit, Birmingham Women's Hospital, University of Birmingham, Birmingham B15 2 TG, UK; 2 Clinical Lecturer in Obstetrics and Gynaecology and Clinical Epidemiology, Academic Unit, 3rd floor, Birmingham Women's Hospital, Birmingham B15 2TG, UK

## Abstract

**Background:**

In maternal medicine, research evidence is scattered making it difficult to access information for clinical decision making. Systematic reviews of good methodological quality are essential to provide valid inferences and to produce usable evidence summaries to guide management. This review assesses the methodological features of existing systematic reviews in maternal medicine, comparing Cochrane and non-Cochrane reviews in maternal medicine.

**Methods:**

Medline, Embase, Database of Reviews of Effectiveness (DARE) and Cochrane Database of Systematic Reviews (CDSR) were searched for relevant reviews published between 2001 and 2006. We selected those reviews in which a minimum of two databases were searched and the primary outcome was related to the maternal condition. The selected reviews were assessed for information on framing of question, literature search and methods of review.

**Results:**

Out of 2846 citations, 68 reviews were selected. Among these, 39 (57%) were Cochrane reviews. Most of the reviews (50/68, 74%) evaluated therapeutic interventions. Overall, 54/68 (79%) addressed a focussed question. Although 64/68 (94%) reviews had a detailed search description, only 17/68 (25%) searched without language restriction. 32/68 (47%) attempted to include unpublished data and 11/68 (16%) assessed for the risk of missing studies quantitatively. The reviews had deficiencies in the assessment of validity of studies and exploration for heterogeneity. When compared to Cochrane reviews, other reviews were significantly inferior in specifying questions (OR 20.3, 95% CI 1.1–381.3, p = 0.04), framing focussed questions (OR 30.9, 95% CI 3.7- 256.2, p = 0.001), use of unpublished data (OR 5.6, 95% CI 1.9–16.4, p = 0.002), assessment for heterogeneity (OR 38.1, 95%CI 2.1, 688.2, p = 0.01) and use of meta-analyses (OR 3.7, 95% CI 1.3–10.8, p = 0.02).

**Conclusion:**

This study identifies areas which have a strong influence on maternal morbidity and mortality but lack good quality systematic reviews. Overall quality of the existing systematic reviews was variable. Cochrane reviews were of better quality as compared to other reviews. There is a need for good quality systematic reviews to inform practice in maternal medicine.

## Background

Maternal medicine has emerged as an increasingly important area for the obstetricians dealing with high risk pregnancies. It involves care of women with medical complications of pregnancy which may be specific to or predate the pregnancy [1]. Approximately half of complex pregnancies are related to an abnormal fetal or obstetric factor, whereas medical diseases constitute the remainder of this high risk obstetric population. Scientific developments in internal or general medicine have led to newer diagnostic and therapeutic strategies to manage medical diseases. The physiological changes during pregnancy can affect not only the clinical presentation of a medical problem but may give rise to difficulties in diagnosing and managing these problems. In order to provide the best possible quality of care to women with complicated pregnancies obstetricians dealing with the high risk obstetric cases should have evidence based knowledge on the diagnostic, therapeutic and prognostic aspects of maternal medicine.

Recently there has been a proliferation of systematic reviews as one of the key tools for evidence-based medicine [2]. As maternal medicine covers the issues related to pregnancy as well as general medicine, research evidence is scattered in the literature making it difficult to access information for clinical decision making. Systematic reviews provide a way forward as individual pieces of research can be collected within literature reviews and if appropriate subjected to meta-analysis [3]. Good methodological quality is essential for these reviews to have valid inferences and to produce usable evidence summaries to guide the obstetric management [4]. This study examines the methodological features of recently published systematic reviews in maternal medicine and specifically compares Cochrane to non-Cochrane reviews.

## Methods

To determine the quality of current systematic reviews in maternal medicine, we developed *a priori *protocol based on recommended methods [2,5-7].

A computerised search of publicly available databases was conducted. Ovid Medline (1996 to date), Embase (1996 to date), Database of Reviews of Effectiveness and Cochrane Database of Systematic Reviews were searched for relevant reviews published between 2001 and 2006. Key word combinations like Pregnan$, Matern$, Gestation$, Obstetric$, Complication$, Systematic review$ and Meta analys$ were used for the search strategy in addition to word variants, subject headings and free text. The $ sign is a truncator used to capture any word that begin with the letters in front of the $ sign in the search terms used. Additionally common and specific medical problems related to pregnancy were searched using key words describing names of the disease such as Pre eclampsia, Hypertension, Diabetes, Cholestasis, Anaemia, Thrombocytopaenia, Thrombophilia, and Thromboembolism. Hand search of reference lists was conducted of all relevant articles to identify any missing reviews. The searches were limited to reviews between 2001 and 2006 due to increasing developments in the field of maternal medicine in recent years. Inclusion criteria required a minimum of two publicly available databases searched for a medical condition specific to or predating pregnancy and maternal factor as the primary outcome. We searched without language restrictions. All the reviews with fetal or neonatal factor as the primary outcome were excluded.

Two reviewers independently extracted and assessed the data according to a checklist formulated as part of our protocol (Table 1). The methodological quality of each review was assessed by focussing on framing of the question, literature search and review methods scrutinising methods of literature search and data synthesis. The items assessed internal validity and explicitness of reporting, both of which are important issues in quality of reviews. Differences between reviewers were resolved by discussion. We computed rates of compliance with the items in our checklist and compared Cochrane and non-Cochrane reviews. Odds ratios and their 95% confidence intervals were computed. All statistical analysis was performed using Stata 8.0 statistical package.

**Table 1:** Checklist used to assess the quality of systematic reviews included in the review.

Title	
Reference	
First Author	
Year of publication	
Journal	
Publication dates of literature included	
No. studies included in the review	
No. of reviewers	
Type of review	therapeutic/prognostic/diagnostic
**Framing of question:**	
Question specified	*yes/no*
Question relevant	*yes/no*
Narrow focus of question	*yes/no*
Explicit testable hypothesis	*yes/no*
**Literature search**	
Adequate search description (incl. names of databases and search terms)	*yes/no*
Use of reference list	*yes/no*
Search without language restriction	*yes/no*
Assessment for risk of missing studies	*yes/no*
Inclusion of unpublished data	*yes/no*
**Quality assessment of included studies:**	
Potential sources of bias (ie. randomisation)	*yes/no*
Data collection (prospective/retrospective)	*yes/no*
Follow-up	*yes/no*
Blinding of assessors*	*yes/no*
Description of intervention*	*yes/no*

*applicable only to interventional reviews

## Results

**Figure 1:**
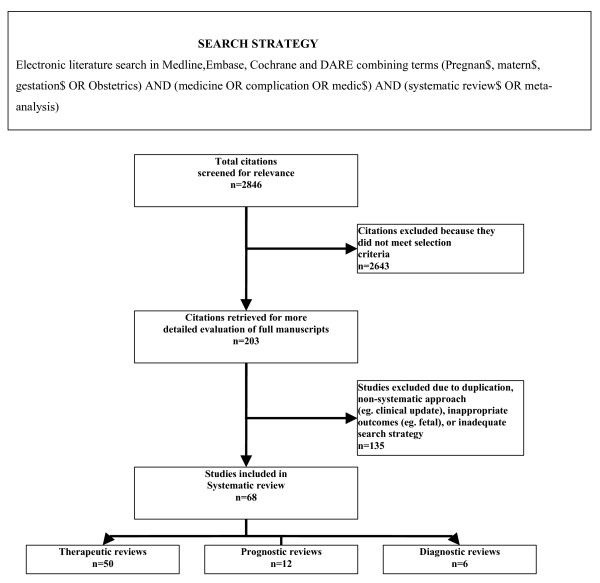
Search strategy and study selection process for review of the methodological features of systematic reviews in maternal medicine.

**Figure 2:**
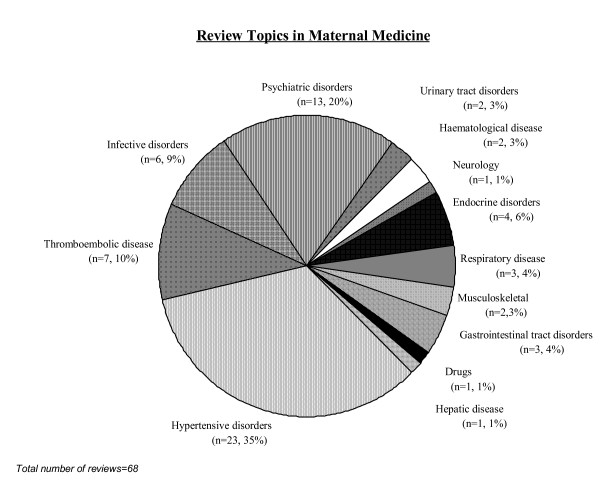
Clinical topics covered by existing maternal medicine reviews.

The initial literature search resulted in 2864 citations. Of these 68 reviews [8-75] fulfilled the inclusion criteria and were selected for detailed study (Fig 1). A total of 39 (57%) Cochrane reviews [8-46] and 29 (43%) non Cochrane reviews [47-75] were included. Most of the reviews assessed therapeutic interventions (50/68, 74%), and the rest were reviews on prognosis (12/68, 17%) and diagnosis (6/68, 9%). The range of clinical topics dealt with by the reviews is shown in Fig 2.

**Figure 3:**
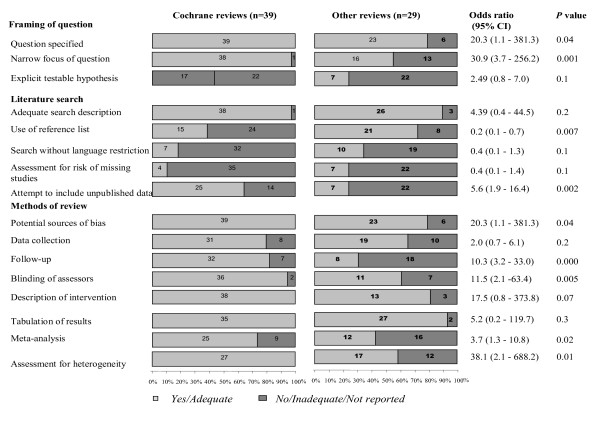
Quality of Cochrane and non Cochrane systematic reviews included in the study.

Overall quality of the existing systematic reviews was variable (Fig 3). Majority of the reviews (62/68, 91%) specified the question and 54/68 (79%) had a focussed question with clearly defined population and outcome measures. A large population of the reviews (64/68, 94%) had a detailed search description including databases searched and key words used. Almost half of the reviews (32/68, 47%) attempted to include unpublished data. However only 11/68 (16%) assessed the risk of missing studies quantitatively and 17/68 (25%) searched without language restriction. Almost all the reviews had good tabulation of results and characteristics of included studies (65/68, 96%).

The quality of Cochrane and non Cochrane reviews is given in Fig 3. Cochrane reviews [8-46] had specified the questions more often than non Cochrane reviews [47-51,53-55,58-60,62-71,73-75] (OR 20.3, 95% CI 1.1–381.3, p = 0.04) and were also framed narrowly focussed questions specifying the population, interventions and comparisons, outcome of the study and the study design (OR 30.9, 95% CI 3.7- 256.2, p = 0.001). Cochrane reviews attempted more often to include unpublished data in literature search (OR 5.6, 95% CI 1.9–16.4, p = 0.002). Twelve out of 29 non-Cochrane reviews [48,50,53-55,64-66,68-71] performed a meta-analysis, but there was good awareness of where this technique was valuable and where it was not applicable. Meta analysis technique (OR 3.7, 95% CI 1.3–10.8, p = 0.02) and assessment for heterogeneity (OR 38.1, 95%CI 2.1, 688.2, p = 0.01) was found to be employed significantly more often by Cochrane reviews.

## Discussion

Our study showed that the Cochrane reviews [8-46] were of consistently high methodological quality and had a greater level of assessment for quality of included studies. They always did a meta-analysis [9,12,17,19-25,27-40,44-46] where applicable. This is reassuring for clinicians who rely on them for decision-making. It is possible that the restriction on the length of published non Cochrane reviews by journals could have influenced their quality scores. However this issue has been addressed by increase in the web publishing of additional material in the electronic format by many journals in recent years.

This work has highlighted that literature searches in reviews are currently generally poor. A search that is not thorough risks giving biased inferences. We identified considerable room for improvement in certain methodological features of non Cochrane reviews. However all the selected reviews were similar in searches without language restriction and assessment for risk of missing studies. Interestingly use of reference list of the selected papers to identify any other eligible studies for inclusion in the review was found to be more frequent in non Cochrane reviews (OR 0.2, 95% CI 0.1–0.7, p < 0.007). This could be a result of the generic search strategy employed by Cochrane reviews with unclear mention of the use of reference lists in individual reviews. Cochrane reviews were found more likely to attempt to include unpublished data compared to non Cochrane reviews (OR 5.6, 95% CI 1.9–16.4, p < 0.002). This attempt to avoid publication bias is significant as the odds of publication are higher if the results are found to be significant compared to studies with non-significant results.[76]

This study identified areas of maternal medicine that lack good quality systematic reviews. Majority of the reviews were on hypertensive disorders [8,10,11,17,19-23,31-34,37,48,53,55,58,64,65,69,70], psychiatry [15,27,43,47,50-52,56,57,59,61,74,75], or thromboembolism [26,42,62-66,68]. Even among these commonly addressed areas, a very narrow spectrum of diseases was covered. For example reviews in psychiatry were solely focussed on depression during pregnancy and reviews in hypertension focussed mainly on pre eclampsia. Reviews for some very common medical problems during pregnancy were missing or of poor quality. We found very few reviews on diabetes mellitus [12,39,49,73] and chronic hypertension and none on thyroid disorders.

With advancement in neonatology and paediatric medicine, more and more women with congenital problems such as congenital heart disease and inherited metabolic diseases are reaching child bearing age and considering pregnancy. There is an urgent need to have some cumulative evidence on management of this high risk group in the best possible way.

This study has some potential limitations. With our strict criteria to include reviews conducted with two publicly available databases, it is possible that some of the good quality reviews in maternal medicine using single database are missed. Another limitation relates to maternal outcome as the main focus of our study. We excluded all those reviews in which association between maternal disease and perinatal outcome was assessed. Keeping in mind the primary goal of an obstetrician being directed towards achieving a healthy and safe outcome for both mother and fetus, good quality evidenced based information on medical problems during pregnancy can only be achieved by reviewing methodological features of all aspects of maternal medicine irrespective of the endpoint. Due to the absence of blinding of the reviewers to the source of the review it is difficult to completely rule out any resultant bias.

## Conclusion

Evidence based healthcare continues to make important contributions to the well being of pregnant women. This study has identified areas in maternal medicine that lack good quality systematic reviews. Overall quality of the existing systematic reviews was variable, with Cochrane reviews better than other reviews. To achieve better understanding and provide high quality obstetric care for pregnant women with medical problems, it is important to ensure that systematic reviews in maternal medicine are conducted to cover wider spectrum of diseases, and are reported at the highest possible quality.

## Competing interests

The author(s) declare that they have no competing interests.

## Funding

None

## Authors' contributions

LS conducted the search, extracted data and drafted the initial version. SJ performed data extraction. ST performed statistical analysis. ST, MK and KSK drafted and approved the final version.

## Pre-publication history

The pre-publication history for this paper can be accessed here:


